# An analytical method informed by clinical imaging data for estimating outlet boundary conditions in computational fluid dynamics analysis of carotid artery blood flow

**DOI:** 10.1038/s41598-023-42004-5

**Published:** 2023-09-11

**Authors:** Muhsin Kizhisseri, Saleh Gharaie, Jorg Schluter

**Affiliations:** School of Engineering, Deakin University, 75 Pigdons Rd, Waurn Ponds, VIC 3216 Australia

**Keywords:** Fluid dynamics, Cardiovascular biology, Computational biology and bioinformatics, Computational models, Computational neuroscience

## Abstract

Stroke occur mainly due to arterial thrombosis and rupture of cerebral blood vessels. Previous studies showed that blood flow-induced wall shear stress is an essential bio marker for estimating atherogenesis. It is a common practice to use computational fluid dynamics (CFD) simulations to calculate wall shear stress and to quantify blood flow. Reliability of predicted CFD results greatly depends on the accuracy of applied boundary conditions. Previously, the boundary conditions were estimated by varying values so that they matched the clinical data. It is applicable upon the availability of clinical data. Meanwhile, in most cases all that can be accessed are arterial geometry and inflow rate. Consequently, there is a need to devise a tool to estimate boundary values such as resistance and compliance of arteries. This study proposes an analytical framework to estimate the boundary conditions for a carotid artery based on the geometries of the downstream arteries available from clinical images.

## Introduction

Atherosclerosis is one of the main reasons for suffering a stroke and it is the world’s major cerebrovascular disease^1^. Atherosclerosis is defined as the build-up of fatty materials on the inner wall lining of arteries which turns into atherosclerotic plaque and eventually results in the cross-sectional area of the arterial lumen diminishing^2^. The prime sites of atherosclerosis inside the arterial lumen are locations with high flow disturbances leading to either low wall shear stress (WSS) regions or unstable oscillatory shear stress regions^3^. The likelihood of plaque formation and atherosclerosis initiation is high for a region with WSS less than 0.4 Pa in the carotid arteries^4–6^. Thus WSS is an important bio marker for determining when atherosclerosis commences, and rupture of cerebral arteries. Arterial geometric factors and blood flow conditions greatly shape the hemodynamic characteristics of blood flow such as blood viscosity, cardiac output, and distal vascular resistance^7^. Complicated and disturbed blood flow is reported in the regions with complex geometry in the cardiovascular systems such as curvatures and bifurcations^8^. Thus the hemodynamics of blood vessels constitute an important aspect of biomedical and biomechanical engineering because the clinical consequences are substantial.

Computational fluid dynamics (CFD) is considered one of the most reliable, non-invasive, and inexpensive approaches in the current scientific environment for understanding the hemodynamics of complex arterial regions such as the carotid artery. It can provide an accurate approximation of velocity, pressure and wall shear stress distributions through a 3D visible representation which is otherwise difficult to obtain by non-invasive techniques^9,10^. CFD simulations can provide better insights into the hemodynamics of regions that are complex and not accessible to physical measurement. Since CFD results have shown good agreement with in vivo results, it is deemed to be the better alternative to physical experiments in studying hemodynamics because of the high cost and time involved in experiments^11,12^. Boundary conditions are very crucial in determining the accuracy of numerical simulations since the selection of boundary conditions wields a high influence on the flow field generated in CFD simulations^13,14^.

Realistic boundary conditions are also one of the prime important factors in the CFD set-up for calculating accurately the pressure, velocity fields and wall shear stress^15,16^. Outlet boundary conditions incorporate hemodynamic characteristics of downstream vascular elements such as capillaries, arterioles, arteries, veins, and venules^15^. Most previous studies give constant velocity, zero pressure, constant pressures, pressure gradient, and constant resistance to the outlet of carotid arteries for convenience. However, the results will not be realistic as they neglect the key distal vascular characteristics, i.e. resistance, impedance, and compliance. The more realistic boundary condition should encompass the natural wave reflections arising from branchings and other geometrical features which are capable of propagating upstream elements. It is also reported that the choice of outflow boundary conditions has important ramifications for the distribution of flow parameters such as pressure field and velocity field along with characteristics of pressure/flow wave propagation^15,17^. Thus, the incorporation of precise outlet boundary conditions plays a prominent role in CFD simulations in providing accurate physiology of blood flow at the carotid artery.

The Windkessel model is a prominent outflow boundary model employed in arterial blood flow simulation studies. It incorporates distal vascular characteristics as outlet boundary conditions into the CFD domain^18–26^. This model is a lumped parameter invention developed by Otto Frank in 1899 to represent the hemodynamics of the arterial network by reproducing physiological relationships between hemodynamic characteristics such as blood flow rate and pressure^24,27^. This lumped parameter model is analogous to an electrical circuit in which arterial blood pressure and flow are represented by electrical voltage and current, respectively^23,28^. The Windkessel model is either represented as 2, 3 or 4-element models in terms of resistance, compliance, impedance and inertance using various electrical circuit analogue elements, for instance resistor, capacitor, and inductor. The arrangement of these elements determines the excellence of the reproduced physiological relationship between blood pressure and flow features in the distal vascular elements of the arterial network, taking into account the vessel properties and flow features^24,27,29^. One of the main advantages of using the Windkessel model is its ability to estimate the reaction of distal vascular elements to potential changes in the blood flow by providing the hemodynamic relationship between pressure and flow waves. It is otherwise very difficult to obtain using invasive pressure measuring techniques in clinical practice^29^.

Pengcheng et al.^9^ conducted an assessment study on different outlet boundary conditions such as the Windkessel model, structured-tree model and fully developed flow model on the carotid artery. They concluded that the Windkessel model appears to be the more accurate and better-performing one, considering all statistical analyses and compared with invasive pressure measurements and Ultrasound Doppler Velocimeter velocity measurements. Olufsen et al. employed a simplified 3-element Windkessel model of blood flow in cerebral arteries to analyse changes in the peripheral and systemic resistance. They asserted that using a simplified lumped parameter model provides easy extraction of dynamic variations of required parameters compared to clinical data. In this way validation is an easier process. They also added that utilising basic models can lead to essential changes in the desired parameters which are otherwise very tedious when using complex lumped parameter models^30^. In a recent study, blood flow in the carotid artery was modelled using the 0D lumped parameter approach in a healthy and stenosed case that depicted a good match between blood flow simulation results and blood flow measurements. This study concluded that analysis of hemodynamics in the carotid artery using the 0D lumped parameter model is an acceptable approach^31^. Another study used a 4-element Windkessel model to assess the blood pressure waveform in the carotid artery for different blood pressure conditions such as normotensive, pre-normotensive and hypertensive. Obtained here were results that matched well with previous studies^23^.

The hemodynamic characteristics such as blood flow rate, blood viscosity and vascular compliance in a cerebrovascular system are also represented by distal vascular resistance. Increasing distal vascular resistance will reduce the blood flow rate in arteries which leads to an increase in the arterial diameter by arterial deformation. Lee et al.^7^ reported that during the systolic phase, the low wall shear stress area increased by 5% for a 20% increase in distal vascular resistance. Transient blood flow simulation conducted on carotid bifurcation reveals that high distal vascular resistance will result in low wall shear stress regions in the carotid artery leading to atherosclerosis progression^37^. Arterial compliance represents the vascular deformation of cerebral arteries in the form of arterial wall motion due to the pulsatile motion of blood flow inside arteries^32–34^. Onaizah et al. discovered that pressure waveforms at the inlet of common carotid artery (CCA) and mean blood flow rate and flow fraction via internal carotid artery (ICA) are impacted by local variations in the carotid artery compliance which in turn affects the supply of blood to the brain. They reported a higher mean blood flow rate at ICA when the compliance in the carotid artery rose when maintaining a fixed flow rate at CCA^35^.

Previous studies have used a tedious and time-consuming process to calculate Windkessel parameters by adjusting resistance and compliance values until they match clinical results. To overcome these challenges, researchers can estimate the Windkessel parameters by mapping the distal vascular elements of the carotid artery, which requires knowledge of the carotid artery's geometrical and anatomical features. However, to date, no research has mapped these features for Windkessel parameter estimation using analytical methods.

This study aims to fill this gap by mapping the distal vascular elements of the internal carotid artery (ICA) and external carotid artery (ECA), calculating the resistance and compliance of each branch and sub-branch of the carotid artery analytically, and validating the results using clinical data through CFD simulations. The values of the Windkessel parameters can serve as initial values for iteration purposes to match hemodynamic parameters measured from CFD studies with clinical data, which can significantly reduce the time spent on iteration purposes.

The data provided in this study can be a great aid to researchers interested in exploring the hemodynamics of the distal cerebral vascular system, as it gives detailed step-by-step values of resistance and compliance at many intersections and divisions along the distal sections of the ACA and MCA. This level of detail is not currently available in the literature and can greatly benefit researchers in the field. The Windkessel parameters for the distal branches of the carotid artery calculated in this study through analytical methods are very relevant in obtaining more accurate CFD results compared to studies that use simplified outlet boundary conditions such as constant pressure, flow splits, and constant velocity. Additionally, this study provides an easier option to establish the resistance and compliance of the distal branches of the carotid artery, which is otherwise a tedious, time-consuming, and computationally costly operation that requires various iterations until matching with clinical results is achieved.

## Methodology

### Geometry and anatomy of different parts of the carotid artery

Carotid arteries are one of the most important parts of the cerebral vascular system since they carry oxygen-rich blood from the heart to the head and brain. The carotid artery originates from the aorta of the heart and is divided into three main regions: the Common Carotid Artery (CCA) which bifurcates into the Internal Carotid Artery (ICA) and the External Carotid Artery (ECA). The branching of the carotid artery takes place in the widening of the carotid artery called a carotid sinus or carotid bulb. ICA supplies blood to the brain whereas ECA supplies blood to the face. Two carotid arteries are located on the left- and right-hand sides of the human neck and are called the Left Carotid Artery and Right Carotid Artery, respectively^36,37^. In this study, We have used information about geometry of different parts of carotid artery and its distal branches from various previously published clinical images and data. No human is directly involved, and no private human data is used for this study.

#### ICA

There are many classifications of the segments of ICA and branches arising from these segments of ICA. According to the recent and most popular classification by Bouthillier et al*.,* ICA is divided into 7 segments and 10 major branches. The segments of ICA are cervical, Petrous, Lacerum, Cavernous, Clinoid, Ophthalmic and Communicating segments, whereas major branches arising from these segments are Caritocotympanic Artery, Vidian Artery, Meningeal Artery, Inferior Hypophyseal Artery, Superior Hypophyseal artery, Ophthalmic Artery, Posterior Communicating Artery, Anterior Choroidal Artery, Anterior Cerebral Artery and Middle Cerebral Artery^38^. In this study, the main focus is on estimating the Windkessel parameters for Middle Cerebral Artery (MCA), Anterior Cerebral Artery (ACA) and the Ophthalmic Artery. The mean length of the ICA reported in one study considering the length from the proximal cavernous segment to the terminus of ICA was 33.1 ± 6.1 mm. The same study reported a mean diameter of 5 ± 0.60 mm and 3.6 ± 0.4 mm at the cavernous and the terminus segments, respectively^39^. MCA and ACA are the terminal branches of ICA.

#### MCA

MCA is the wider terminal branch of the ICA which extends from the base to the lateral surface of the brain through the lateral sulcus of Sylvius supplies blood to the lateral cerebral hemisphere, insula, temporal pole, lentiform nucleus, internal capsule and other deep structures of the brain^40^. Based on the anatomic structures and surgical classifications, MCA is divided into four segments which extend in a step-by-step angular manner: M1 (sphenoidal segment), M2 (insular segment), M3 (opercular segment), and M4 (cortical segment). There are 10 main branches arising out of these segments and they are as follows: lateral lenticulostriate arteries, anterior temporal arteries, orbitofrontal arteries, prefrontal arteries, precentral arteries, central arteries, postcentral arteries, parietal arteries, angular arteries and middle temporal arteries^40^^,^^41^.

The M1 segment originates in the Sylvian cistern at the terminal bifurcation of the ICA travelling parallel to the sphenoid ridge inside the proximal Sylvian fissure^40^^,^^42^. One research study reported the mean diameter of M1 as 3.49 mm^43^. Reci et al. reported variations in the length, shape and branching of M1 segment in 50 different specimens. As per this study, 64% of M1 segments are arch-shaped with an average length and diameter of 19.8 ± 4.41 mm and 2.6 ± 0.47 mm respectively. Conversely, 24% is S-shaped with proportions of 20.6 ± 3.31 mm (length), 2.7 ± 0.51 mm (diameter) and 12% is straight-shaped with a length of 16.9 ± 3.62 mm and diameter of 2.6 ± 0.36 mm^44^. Average length and diameter for the M1 segment were calculated based on information from the above article, i.e. 19.644 mm in length and 2.664 mm in diameter.

The major branches of the M1 segment are anterior temporal arteries (ATA) and lateral lenticulostriate arteries (LSA). Both arteries are located on opposite sides of the M1 segment with lenticulostriate arteries on the front side and ascend before M2s bifurcation whereas ATA is on the rear side^40^. The LSAs are generally 6–12 in number with each branch diameter ranging from 0.08 mm to 1.4 mm with a mean diameter of 0.47 mm. The average length of LSAs is 21.9 mm^45^. In this study, LSAs are assumed to be 9 in total considering the average number of branches. The diameter of ATAs ranges from 1.5 to 2 mm with an average value of 1.75 mm as applied in this study^46^.

Previous studies show that 78% of the M1 segment bifurcates into superior and inferior trunks of M2 segments, while 12% trifurcates into superior, inferior, and middle trunks of M2 segments. The remaining 10% divides into various smaller superior and inferior terminal branches. These bifurcations and trifurcations exist mainly at the insular cistern after M1 makes a sharp turn around the front end of the limen insula resulting in M2 branches located in the frontal end of the insula. Major superior terminal branches are the prefrontal sulcal artery, lateral frontonasal artery, Rolandic (central) sulcal and pre-Rolandic (precentral) artery. Major inferior terminal branches are the temporal artery, angular artery and parietal arteries^40^^,^^42^. In this study, M1 bifurcates into two trunks of M2 with ATA added along with the bifurcation since it is located towards the rear of the M1–M2 intersection. The M2 segments generally have 8–12 branches^40^, but in this study the majority of those branches are neglected as their size is very negligible. In addition, the lack of authentic geometric measurements of these branches also forced us to neglect these. One study reported M2 branches as having an average diameter of 2.4 ± 0.4 mm^39^ whereas another analysis documented mean diameter of M2 ranging from 1.4 to 2.3 mm, and the mean length varied from 12.1 to 14.9 mm^47^. In this study, M2 diameter and length are assumed to be 2.4 mm and 13.5 mm, respectively, taking in account popular opinion and average values noted in previous studies.

The M2 segments after taking an oblique turn through the opercular extension of the Sylvian fissure form the M3 segment of MCA. An accurate geometric estimation of the length and diameter of M3 has not yet been correctly reported. In that case, in this study, an average diameter of 1.7 mm and length of 13.3 mm is assumed for the M3 segment considering the mean diameter and length of adjacent segments M2 and M4. M3 segments after taking a right angle turn forms the M4 segments in the cortex region. One study reported the diameter of the cortical segment as 1 mm with segment length ranging from 0 to 40 mm with a mean length of 13.1 mm^48^. It is reported that 6 to 13 branches arise from the M4 cortical segment which includes orbitofrontal, prefrontal, precentral, central, temporopolar, anterior temporal, middle temporal, posterior temporal, and temporo-occipital, anterior parietal, angular and posterior parietal arteries^49^.

In this study, 3 main branches of the M4 segment are considered and they are the lateral orbitofrontal artery (LOFA), angular artery and temporopolar artery. These branches are considered major ones in cortical segments in many studies^47,48^. The angular artery is the largest vessel in the M4 segment on both sides of the brain with a mean diameter of 1.5 mm whereas the temporopolar artery is the smallest with a mean diameter of 0.8 mm and 0.9 mm on the right and left hemispheres, respectively^47^. In this study, an average value of 0.85 mm is assumed for the temporopolar artery. The lateral orbitofrontal artery is found to range from 2 to 40 numbers with an average number of 15.78 ± 10.44 for supply to the lateral orbitofrontal cortex^48^. In this paper, 16 branches of LOFA are considered. A previous study reported an average diameter of 0.8 mm for LOFA from a range of 0.4–1.5 mm and an average length of 13.1 mm from a range of 0–40 mm^48,50^. In this study, both angular artery and temporopolar artery are assumed to have the same segment length of LOFA due to the lack of credible medical data.

#### ACA

The anterior cerebral artery (ACA) is also a terminal branch of the ICA which supplies blood to the interhemispheric region. The ACA is mainly divided into two parts called proximal parts (A1) and distal parts (A2 to A5)^51^. ACA segment before and after the anterior communicating artery (AComA) is known as the proximal parts and distal parts, respectively. The distal section of ACA contains cortical and central branches and is also called the pericallosal artery^51,52^. The mean diameter of ACA at origin was reported as 2.61 ± 0.34 mm while its mean length from vessel origin to AComA is 7.68 ± 3.91 mm in the orbitofrontal region. Conversely, 112.6 ± 11.63 mm in the inferior internal parietal region was reported^52^. In this study, the mean length of ACA in the orbitofrontal region is noted. ACA is mainly divided into 5 segments, i.e. A1–A5. A1 segment is also called a pre-communicating or horizontal segment. It commences from the terminal bifurcation of ICA with a segment length of 14 mm and mean diameter of 2.6 mm^51^.

The main branches that arise from the A1 segment are AComA and medial lenticulostriate arteries^53^. Sousa et al.^51^ reported that the AComA arises from the terminals of the A1 segment with a mean diameter of 1.6 mm and a mean length of 2–3 mm; however, the length of AComA varies from 0.3 to 7 mm. In another study, the exposed length of ACA is determined as 3 ± 2 mm and 3.3 mm^54,55^. Considering the mean values in both studies, in this study the mean length of AComA is 3 mm. The AcomA contains perforating branches called Subcallosal-Hypothalamic Perforating Branches and their average number is 7.6 (range from 4 to 15) and a diameter of 0.4 ± 0.2 mm (0.1–1 mm range)^56^. The median callosal artery also originates from AComA with a mean diameter of 0.75 mm (range 0.5–1 mm)^57^. The length of these perforating branches and arteries in the AcomA is assumed to be 5 mm when the geometric features of nearby arteries are considered. The medial lenticulostriate arteries are 2 to 15 in number and are reported to be shorter in length and thinner in diameter than lateral lenticulostriate arteries in MCA^58^. In this study, they have a diameter of 0.4 mm, length of 20 mm and number 9 in total.

The A2 segment which starts at the AComA ranges through the frontal portion of lamina terminalis laterally to the corpus callosum rostrum, and terminates at the beginning of callosimarginal artery or corpus callosum genu. A2 is known as either the infracallosal, post-communicating or vertical segment of ACA. The main branches that arise from the A2 segment are the recurrent artery of Heubner (RAH), orbitofrontal artery (OFA) and frontopolar artery (FPA)^53^. The RAH which is also known as the distal medial striate artery originates either from the proximal part of A2 or the distal portion of A1; it has a mean length and radius of 24 mm and 0.7 mm (range is 0.2–2.9 mm), respectively^51,59^. The orbitofrontal arteries in the ACA segment are called medial orbitofrontal arteries (MOFA) with a mean number of 15 branches and a range of 2–28 branches in the ACA. The MOFA is located 7.9 mm anterior to the AComA with a mean diameter of 1 mm and a range of 0.2–2 mm^48^. The FPA is reported to have an average diameter of 1.02 ± 0.46 mm and an average length of 10.7 ± 5.1 mm from the beginning of FPA to AComA^60^.

The A3 segment of ACA is called the precallosal segment which is positioned distal to the origin of the callosomarginal artery (CMA), extending and turning around the corpus callosum genu and terminating above the rostral part of the corpus callosum^51,53^. The CMA is considered one of the prominent branches in the distal part of the ACA with a diameter ranging from 1.8 to 1.9 mm^51^. In this study, a mean diameter of 1.85 mm is established for CMA. The A4 segment of ACA is called a supracallosal segment which is positioned above the frontal portion of the corpus callosum body to the level of the coronal structure. Conversely, the A5 segment is called a post-callosal segment which is positioned above the latter portion of the corpus callosum body to the level of coronal structure^53^. The major branches arising from both these segments are the paracentral artery, inferior and superior parietal artery and posterior internal frontal artery^51^. Major studies report that it is very hard to determine the geometric parameters of distal and cortical branches of arteries from A3 to A5 segments because of the high variations in the origin, and diameter of these distal segments and branches^51^. However, a study reported the diameter of cortical branches ranges from 0.79 ± 0.27 mm to 1.84 ± 0.3 mm^53^. In this scenario, this study has given an assumed value for the diameters of A3, A4 and A5 as 2, 1.5 and 1 mm, respectively. Zhu et al.^56^ found the average total length of the distal segment of ACA is 250 mm. Considering this measurement and eliminating the length of A1 and A2 segments and other branches along with the distal segments, the A3, A4 and A5 segments are each assumed to be 30 mm in length.

#### Ophthalmic artery

The ophthalmic artery is one of the smallest branches arising from the ICA which supplies to the eye orbit and nearby structures. This artery extends through the orbital cavity and has 10 branches along its course. The branches of the ophthalmic artery are the central retinal artery, posterior ciliary arteries, muscular branches, lacrimal artery, medial palpebral arteries supraorbital artery, posterior and anterior ethmoidal artery, dorsal nasal artery, and supratrochlear artery^61^. Studies report that the central retinal artery has an average diameter and length of 0.16 mm and 7.5 ± 2.2 mm, respectively^62,63^. Considering the length of the central retinal artery, the posterior ciliary artery is given an assumed diameter of 0.1 mm, whereas the mean length of this artery is reported to be in the 3–7 mm range^64^. The remaining branches of the ophthalmic artery is not examined in this study as their geometrical parameters are too small to have any impact on the results of this study.

#### ECA segments

The main branches of ECA are the Superior thyroid artery, Lingual artery, Occipital artery, Maxillary artery, Ascending pharyngeal artery, Posterior auricular artery, Superficial temporal artery, and Facial artery^65^. Arjun et al*.* measured the diameter of the superior thyroid artery (STA) as 2 mm whereas in another study the diameter was 0.5–1.5 mm and the length 30 mm^66–68^. The ascending pharyngeal artery (AP) is located in the frontal position of the proximal side of the ECA with an average diameter of 1 mm and length of 70 mm^67,69^. The lingual artery is reported to have a diameter of 2–5 mm and a length which is approximately 75% that of the dorsal portion to the tip of the human tongue^70,71^. The mean length of the human tongue is 90 mm, so in this study, the length of the lingual artery is noted as 67.5 mm^72^. The mean diameter of the facial artery at the origin and anteroinferior angle of masseter is within the 3.0–3.2 mm range for both males and females whereas the mean length is 28 mm^67,73,74^. Ozkan et al.^75^ measured the mean thickness and length of the occipital artery (OA) as 1.9 mm and 79.3 mm, respectively. A study reported the range of diameter and length of the posterior auricular artery as 0.7–1.2 mm and 64 ± 13 mm, respectively^76^. The maxillary artery is estimated to have an average diameter of 4.08 ± 0.51 mm at the location of its branching from ECA and an average length of 80 mm^67,77,78^. Marano et al.^79^ reported the average diameter and length of the superficial temporal artery as 2.2 mm and 70 mm, respectively. Hiller et al.^68^ measured the averaged diameter of the proximal and distal ECA as 4 ± 0.6 mm and 2.85 ± 0.4 mm, respectively.

### Windkessel parameter calculations of carotid artery segments

The Windkessel model is a zero-dimensional (0-D) one and it is analogous to the electrical circuit. Based on the mathematics of lumped parameter model,

1 $${\text{Hydraulic Resistance}}\quad R = \frac{128\mu l}{{\pi D^{4} }}$$

2 $${\text{Elastic Capacitance of blood vessel }}\left( {{\text{compliance}}} \right)\quad C = \frac{{\pi D^{3} l}}{4Gh}$$

whereas $\mu$ is the blood viscosity, $l$ denotes the segment length, $D$ is the diameter of the artery segment, $G$ stands for Young’s modulus of the blood vessel and $h$ represents the wall thickness of the blood vessel. Resistor with resistance (R) represents viscous friction experienced by the blood flow in the arterial system, especially at the blood-vessel interface of small arteries and arterioles. A resistor is arranged in two ways, either as proximal resistance (R_p_) or distal resistance (R_d_). Proximal resistance represents the viscous resistance of the proximal regions of the vasculature whereas distal resistance represents the resistance of the distal regions such as capillaries, arterioles, and venous circulation. The capacitor with capacitance (C) represents the compliance or elasticity of blood vessels^17,23–26,80^. In this study, we are considering a three-element Windkessel model (RCR) with proximal resistance (R_p_), compliance (C) and distal resistance (R_d_). The blood flow rate and pressure distribution at the outlet of the CFD model of the carotid artery using the RCR Windkessel model are shown by a partial differential equation (Eq. 3), which explains the relationship between current and voltage in the electrical circuits:

3 $$Q\left(1+\frac{{R}_{P}}{{R}_{d}}\right)+C{R}_{P}\frac{\partial Q}{\partial t}=\frac{P}{{R}_{d}}+C\frac{\partial P}{\partial t}$$

**Figure 1:**
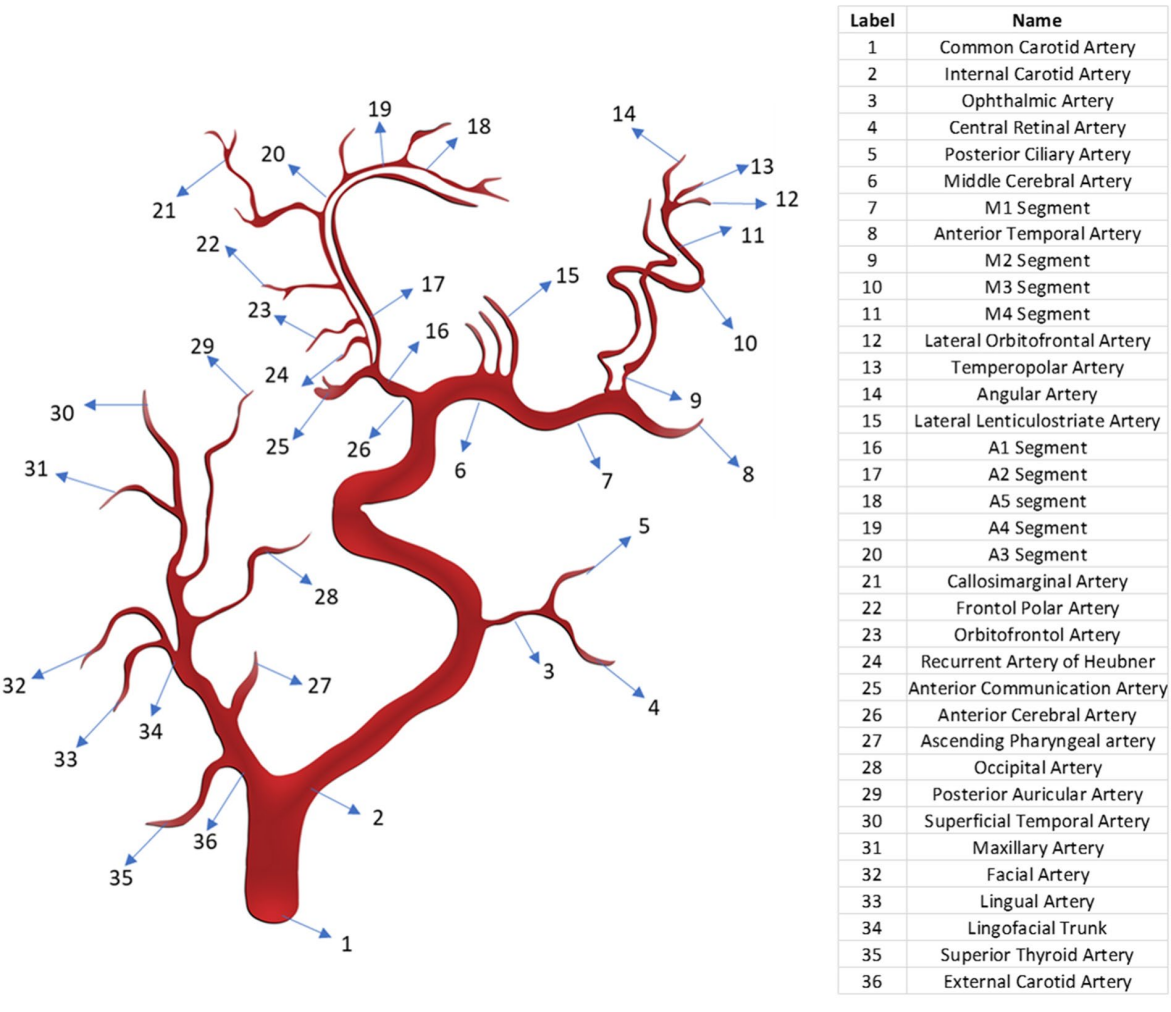
Mapping of distal vascular elements of the carotid artery.

In Eq. (3), $Q$ represents the blood flow rate which is similar to the current in the electrical circuits, whereas $P$ represents the time-dependent blood pressure which is analogous to voltage in the electrical circuit. In this study, resistance R and compliance C of all the branches and sub-branches of the carotid artery are analytically calculated by using Eqs. (1 and 2). The results are listed in Table 1. The total proximal resistance, distal vascular resistance and compliance of the ICA and ECA branches were calculated based on the arrangement of the branches and sub-branches of the carotid artery in Fig. 1. Based on this arrangement, the decision is made to choose the series and parallel arrangement of the resistance and compliance of the carotid artery’s distal vascular elements. The total resistance and compliance of each branch are estimated based on the equivalent resistance and compliance in series or parallel conditions which are shown in Tables 2, 3, 4 and 5. In the next step, a CFD simulation was done to validate the analytically calculated values of resistance and compliance of branches of the CCA.

**Table 1:** Resistance and compliance of carotid artery branches and sub-branches.

Label	Part/segment/branch	Diameter (mm)	Length (mm)	Nos	Resistance (kg m^−4^ s^−1^)	Compliance (kg^−1^m^4^ s^2^)
B1	ICA (proximal segment)	5.6	33.1	1	4.80E + 06	1.48E−12
B2	M1 (sphenoidal segment)	2.66	19.6		5.60E + 07	1.07E−13
B3	Lateral lenticulostriate artery (LLS)	0.47	21.9	9	6.40E + 10	6.60E−16
B4	M2 (insular segment)	2.4	13.5		5.81E + 07	5.42E−14
B5	Anterior temporal artery (ATA)	1.75	10		1.52E + 08	1.56E−14
B6	M3 (opercular segment)	1.7	13.3		2.27E + 08	1.90E−14
B7	M4 (cortical segment)	1	13.1		1.87E + 09	3.80E−15
B8	Angular artery	1.5	13		3.66E + 08	1.27E−14
B9	Temporopolar artery	0.85	13		3.55E + 09	2.32E−15
B10	Lateral orbitofrontal artery (LOFA)	0.8	11.1	16	3.87E + 09	1.65E−15
B11	Anterior cerebral artery (Origin)	2.61	7.6		2.36E + 07	5.11E−14
B12	RAH(A2)	0.7	24		1.43E + 10	2.83E−15
B13	Anterior cerebral artery (Cortical segment)	1.31	10		4.77E + 08	8.51E−15
B14	A1 (horizontal or pre-communicating segment)	2.5	20		7.30E + 07	1.17E−13
B15	A2 (infracallosal segment)	3	42.1		7.42E + 07	3.91E−13
B16	A3 (precallosal segment)	2	30		2.68E + 08	6.13E−14
B17	A4 (supracallosal segment)	1.5	30		8.45E + 08	3.79E−14
B18	A5 (postcallosal segment)	1	30		4.28E + 09	1.12E−14
B19	Anterior communication artery	1.6	3		6.53E + 07	4.60E−15
B20	Medial lenticulostriate artery	0.4	20	9	1.11E + 11	3.27E−16
B21	Callosomarginal artery	1.85	10		1.22E + 08	2.18E−14
B22	Medial orbitofrontal artery (MOFA)	1	7.9	15	1.13E + 09	2.72E−15
B23	Frontopolar artery	1.02	10.7		1.41E + 09	3.90E−15
B24	Subcallosal-hypothalamic perforating branches	0.4	5	7.6	2.79E + 10	1.10E−16
B25	Median callosal artery	0.75	5		2.25E + 09	7.25E−16
B26	Central retinal artery	0.16	7.5		1.63E + 12	1.03E−18
B27	Posterior ciliary artery	0.1	5		7.13E + 12	1.68E−19
B28	Ophthalmic artery	–	–	–	1.33E + 12	1.20E−18
B29	Superior thyroid artery	2	30		2.68E + 08	8.09E−14
B30	Ascending pharyngeal artery	1	70		9.99E + 09	2.36E−14
B31	Lingual artery	2.5	67.5		2.47E + 08	3.55E−13
B32	Facial artery (f)	3.2	28		3.81E + 07	3.09E−13
B33	Occipital artery (o)	1.9	79.3		8.68E + 08	1.83E−13
B34	Posterior auricular artery (pa)	0.95	64		1.12E + 10	1.85E−14
B35	Maxillary artery (m)	4.08	80		4.12E + 07	1.83E−12
B36	Superficial temporal artery (stm)	2.2	70		4.26E + 08	2.51E−13
B37	Proximal ECA main branch segment	4	20		1.11E + 07	4.31E−13
B38	Distal ECA main branch segment	3	20		3.52E + 07	1.82E−13

**Table 2:** Resistance calculation for ICA segment based on the arrangement of distal branches/sub-branches.

Label	Resistance (kg m^−4^ s^−1^)	Explanation
R1	1.40E + 08	Total resistance of the B8, B9 and B10 in parallel condition for distal end of M4
R2	2.01E + 09	Total resistance of R1 and B7 in series condition for M4
R3	2.29E + 09	Total resistance of R2, B6 and B4 in series condition
R4	1.34E + 08	Total resistance of 2*R3 and B5 in parallel condition for M1–M2 intersection
R5	7.11E + 09	Total resistance of 9*B3 in parallel condition for LLS branches
R6	5.55E + 07	Total resistance of R5 and B2 in parallel condition for M1 segment
R7	1.90E + 08	Total resistance of R4 and R6 for MCA branch
R8	5.39E + 09	Total resistance of B16, B17 and B18 in series condition for Distal segment of ACA
R9	7.51E + 07	Total resistance of 16*B22 in parallel condition for MOFA branches
R10	7.10E + 07	Total resistance of R9, B23 and B12 in parallel condition for branches in A2 segment
R11	3.63E + 07	Total resistance of R10 and B15 in parallel condition for A2 segment
R12	5.43E + 09	Total resistance of R11 and R8 in series condition
R13	2.71E + 09	Total resistance of 2*R12 in parallel condition for segments from A2 till A5
R14	3.67E + 09	Total resistance of 7.6*B24 in parallel condition for Subcallosal-Hypothalamic Perforating branches
R15	1.40E + 09	Total resistance of R14 and B25 in parallel condition for AComA branches
R16	6.24E + 07	Total resistance of R15 and B19 in parallel condition for AComA segment
R17	6.17E + 07	Total resistance of R12 and P16 in parallel condition at A1-AComA-A2 intersection
R18	1.35E + 08	Total resistance of B14 and R17 in series condition for ACA segment
R19	5.89E + 07	Total resistance of R7 and R18for distal ICA segment

**Table 3:** Compliance calculation for ICA segment based on the arrangement of distal branches/sub-branches.

Label	Compliance (kg^−1^m^4^ s^2^)	Explanation
C1	1.67E−14	Total compliance of the B8, B9 and B10 in parallel condition
C2	3.10E−15	Total compliance of C1 and B7 in series condition
C3	2.54E−15	Total compliance of C2, B6 and B4 in series condition
C4	2.06E−14	Total compliance of 2*R3 and B5 in parallel condition for M1–M2 intersection
C5	5.94E−15	Total compliance of 9*B3 in parallel condition for LLS branches
C6	1.13E−13	Total compliance of R5 and B2 in parallel condition for M1 segment
C7	1.75E−14	Total compliance of R4 and R6 for MCA branch
C8	7.59E−15	Total compliance of B16, B17 and B18 in series condition for Distal segment of ACA
C9	4.07E−14	Total compliance of 16*B22 in parallel condition for MOFA branches
C10	4.75E−14	Total compliance of R9, B23 and B12 in parallel condition for branches in A2 segment
C11	4.38E−13	Total compliance of R10 and B15 in parallel condition for A2 segment
C12	7.46E−15	Total compliance of C11 and C8 in series condition
C13	1.49E−14	Total resistance of 2*C12 in parallel condition for segments from A2 till A5
C14	8.36E−16	Total compliance of 7.6*B24 in parallel condition Subcallosal-Hypothalamic Perforating Branch
C15	1.56E−15	Total compliance of C14 and B25 in parallel condition for AcomA branches
C16	6.16E−15	Total compliance of C15 and B19 in parallel condition for AComA segment
C17	1.36E−14	Total compliance of C12 and C16 in parallel condition at A1-AComA-A2 intersection
C18	1.22E−14	Total compliance of B14 and C17 in series condition for ACA segment
C19	3.79E−14	Total compliance of C7 and C18 for distal ICA segment

**Table 4:** Resistance calculation for ECA segment based on the arrangement of distal branches/sub-branches.

Label	Resistance (kg m^−4^ s^−1^)	Explanation
R20	3.76E + 07	Total resistance of B36 and B35 in parallel condition
R21	7.28E + 07	Total resistance of R20 and B38 in series condition
R22	7.23E + 07	Total resistance of R21 and B34 in parallel condition
R23	1.08E + 08	Total resistance of R22 and B38 in series condition
R24	9.57E + 07	Total resistance of R23 and B33 in parallel condition
R25	1.07E + 08	Total resistance of R24 and B37 in series condition
R26	3.30E + 07	Total resistance of B31 and B32 in parallel condition
R27	2.52E + 07	Total resistance of R25 and R26 in parallel condition
R28	3.64E + 07	Total resistance of R27 and B37 in series condition
R29	3.19E + 07	Total resistance of R28, B29 and B30 in parallel condition for the distal resistance of ECA segment

**Table 5:** Compliance calculation for ECA segment based on the arrangement of distal branches/sub-branches.

Label	Compliance (kg^−1^m^4^ s^2^)	Explanation
C20	2.08E−12	Total compliance of B36 and B35 in parallel condition
C21	1.67E−13	Total compliance of C20 and B38 in series condition
C22	1.86E−13	Total compliance of C21 and B34 in parallel condition
C23	9.19E−14	Total compliance of C22 and B38 in series condition
C24	2.75E−13	Total compliance of C23 and B33 in parallel condition
C25	1.68E−13	Total compliance of C24 and B37 in series condition
C26	6.64E−13	Total compliance of B31 and B32 in parallel condition
C27	8.32E−13	Total compliance of C25 and R24 in parallel condition
C28	2.84E−13	Total compliance of C27 and B37 in series condition
C29	3.89E−13	Total compliance of C28, B29 and B30 in parallel condition for the distal resistance of ECA segment

### CFD simulations and validation

In this study, validation of the analytical results on the Windkessel parameters was conducted by using computational fluid dynamics (CFD) simulations on two different geometries of carotid artery.

1. A CFD simulation was performed on a simplified model of the carotid artery, utilizing the resistance and compliance values for the proximal sections of the internal carotid artery (ICA) and external carotid artery (ECA). The primary objective was to determine whether the analytically calculated Windkessel parameters accurately reflected the flow rate splits between ICA and ECA, in comparison to the generalized clinical flow split ratio.
2. A CFD simulation was carried out on a patient-specific geometry of the ICA, with a particular emphasis on the distal sections including branches such as the Ophthalmic artery, Anterior Cerebral Artery (ACA), and Middle Cerebral Artery (MCA) segments (M1 and M2). This simulation incorporated the analytically calculated resistance and compliance values for these distal branches. The CFD results were then compared to the phase-contrast magnetic resonance imaging (PCMRI) data obtained from the patient. The PCMRI data included blood flow velocity measurements from the MCA and ACA after the ICA bifurcation.

#### CFD simulations on a simple carotid artery model

**Figure 2:**
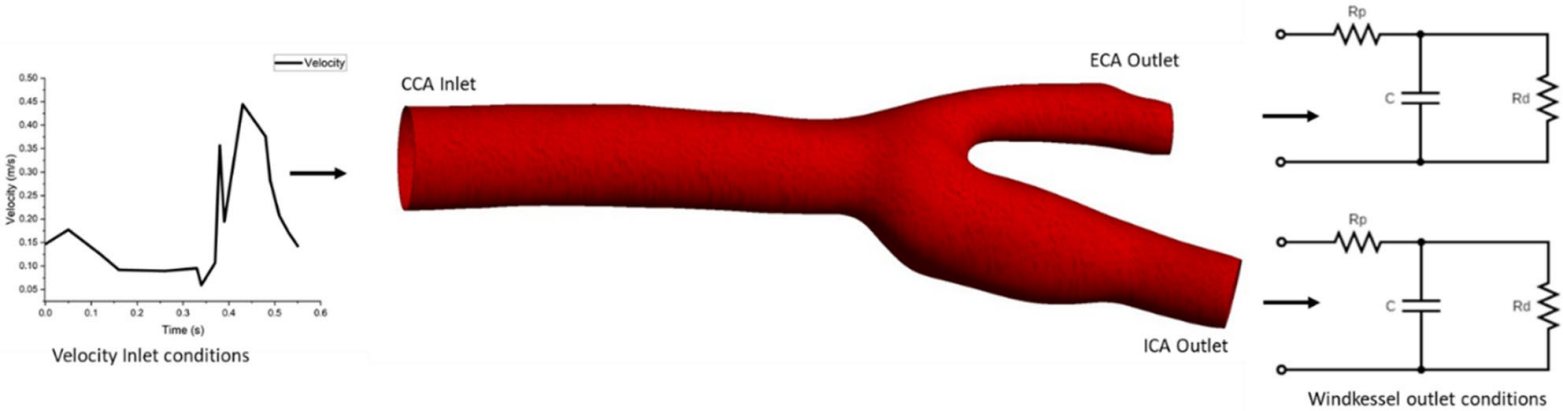
CFD model for simplified carotid artery used in this study.

A CFD simulation was done on the carotid artery geometry to validate the results obtained in this study. The Windkessel parameters calculated in this study for both ICA and ECA are applied to this geometry and monitored the flow splits between ICA and ECA. The CFD simulation was done using the commercial software ANSYS Fluent 2022 b. A 3D model of the carotid artery provided by ANSYS Inc. was employed in this study. Meshing was done using Ansys meshing in the Workbench 2022 b version. The tetrahedron grid element was used in the meshing with a minimum element size of 0.4 mm which has a finer mesh with 294,255 mesh elements and 92,840 nodes. This mesh was chosen after conducting the mesh refinement study. Inflation layers were also given to the boundaries of the wall to enable refinement near the wall. Blood flow is assumed to be an incompressible laminar flow with Newtonian characteristics. Blood flow density is given as 1060 kg/m^3^ and viscosity as 0.004 Pa.s. A clinical blood flow rate was given as the inlet boundary condition which is obtained from one study^81^. This inlet blood flow rate was given to the CFD domain as a form of User Defined Function (UDF). The Windkessel-based outlet boundary conditions were given to the model based on analytically calculated values of the parameters resistance and compliance based on the results in Tables 1, 2, 3, 4, 5. The simulation time of the one cardiac cycle is given as 0.55 s based on the clinical data obtained from^81^ with an initial time step size of 0.001S. The simulation was done using the adaptive method with the solution convergence criteria of 10^−6^ for all variables. The simulation was done for 7 cardiac cycles and the blood volume flow rate split between the ICA and ECA branches is monitored from the CFD simulations. This captures the blood flow ratio of ICA and ECA with respect to the CCA blood flow rate. Figure 2 gives the representation of CFD model used in this study.

#### CFD simulations on a patient specific internal carotid artery model

In this approach, a patient-specific 3D model of the internal carotid artery and PCMRI clinical results, provided by See-Mode Technologies Pte. Ltd., was utilized. The computational fluid dynamics (CFD) simulation was conducted using the commercial software ANSYS Fluent 2022 b. The meshing process was performed using Ansys Meshing in the Workbench 2022 b version. The tetrahedral grid element was employed, with a minimum element size of 0.3 mm, resulting in a finer mesh consisting of 431,468 mesh elements and 177,603 nodes. This mesh configuration was selected following a mesh refinement study. Inflation layers were added to the boundaries of the wall to allow for refinement near the wall.

**Figure 3:**
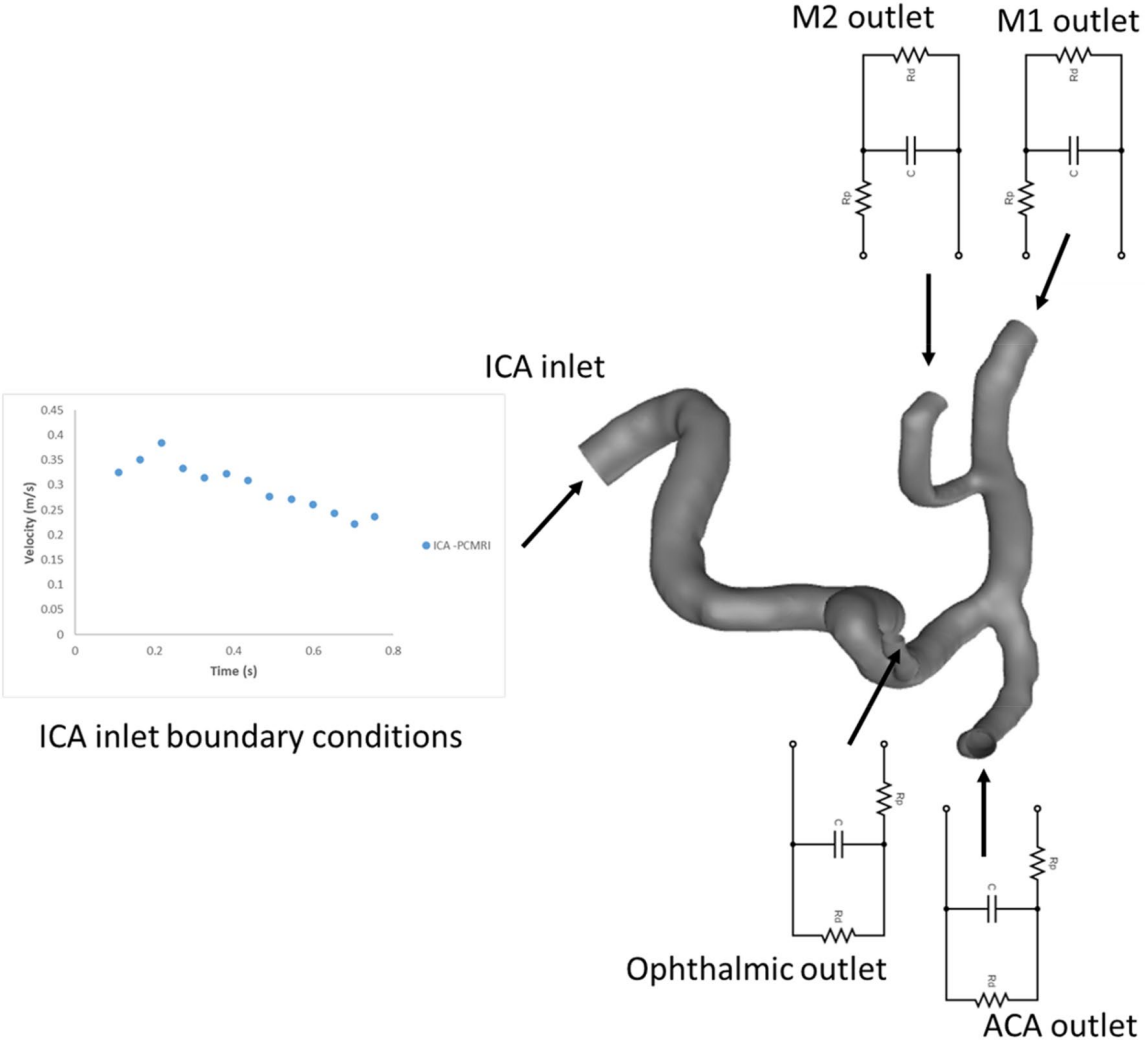
CFD model used for patient specific internal carotid artery.

The blood flow within the model was assumed to be incompressible, laminar, and exhibit non-Newtonian characteristics. The Carreau model was employed to incorporate these non-Newtonian properties, with the following parameter values: time constant of 3.313 s, Power-Law index of 0.3568, Zero Shear Viscosity of 0.056 kg/(ms), and Infinite Shear Viscosity of 0.0035 kg/(ms). The blood flow density was set to 1060 kg/m^3^. The patient-specific blood flow velocity measurements obtained from the internal carotid artery using the PCMRI technique were assigned as the inlet boundary condition. This inlet blood flow rate was implemented in the CFD domain using a User Defined Function (UDF). The outlet boundary conditions of the model, such as M1, M2, ACA, and the Ophthalmic artery, were defined based on analytically calculated values of the parameters resistance and compliance, as presented in Tables 1, 2, 3, 4, 5. The simulation time for one cardiac cycle was determined as 0.753 s, based on the PCMRI data, with an initial time step size of 0.01 s. The simulation was conducted using the adaptive method, with a solution convergence criterion of 10^−6^ for all variables. The simulation was performed over five cardiac cycles. Figure 3 gives the representation of CFD model used in this study.

## Results

In this study, resistance R and compliance C of all the branches and sub-branches of the carotid artery are analytically calculated using Eqs. (1 and 2); the results are listed in Table 1. The total proximal resistance, distal vascular resistance and compliance of the ICA and ECA branches were calculated based on the arrangement of the branches and sub-branches of the carotid artery in Fig. 1. Based on this arrangement, the decision is made on choosing the series and parallel arrangement of resistance and compliance of the carotid artery’s distal vascular elements. The total resistance and compliance of each branch are estimated based on the equivalent resistance and compliance in series or parallel conditions which are shown in Tables 2, 3, 4, 5. The proximal segment of the ICA has a resistance of 4.80E + 06 kg m^−4^ s^−1^ and compliance of 1.48E−12 kg^−1^m^4^ s^2^. The analytical method used calculating the compliance and resistance of the distal branches of ICA is shown in Tables 2 and 3. The total distal vascular resistance and compliance of the ICA branch are 5.89E + 07 kg m^−4^ s^−1^ and 3.79E−14 kg^−1^m^4^ s^2^, respectively. Referring to the ECA branch, Tables 4 and 5 show the analytical calculation method used for resistance and compliance. The proximal resistance and compliance of the ECA branch are 1.11E + 07 kg m^−4^ s^−1^ and 4.31E−13 kg^−1^m^4^ s^2^, respectively. The total distal vascular resistance and compliance of the ECA branch are 3.19E + 07 kg m^−4^ s^−1^ and 3.89E−13 kg^−1^m^4^ s^2^, respectively.

The aim of the first validation study is to calculate the volumetric flowrate split ratio using resistance and compliance as the outlet boundary conditions through the Windkessel model for the ECA and ICA outlets and compare it with clinical results. This is a crucial part of the validation process as the flow split ratio is sensitive to changes in outlet boundary conditions. If the calculated flow split ratio from CFD studies using these parameters matches clinical results, it demonstrates that the outlet boundary conditions given in this study are accurate.

**Figure 4:**
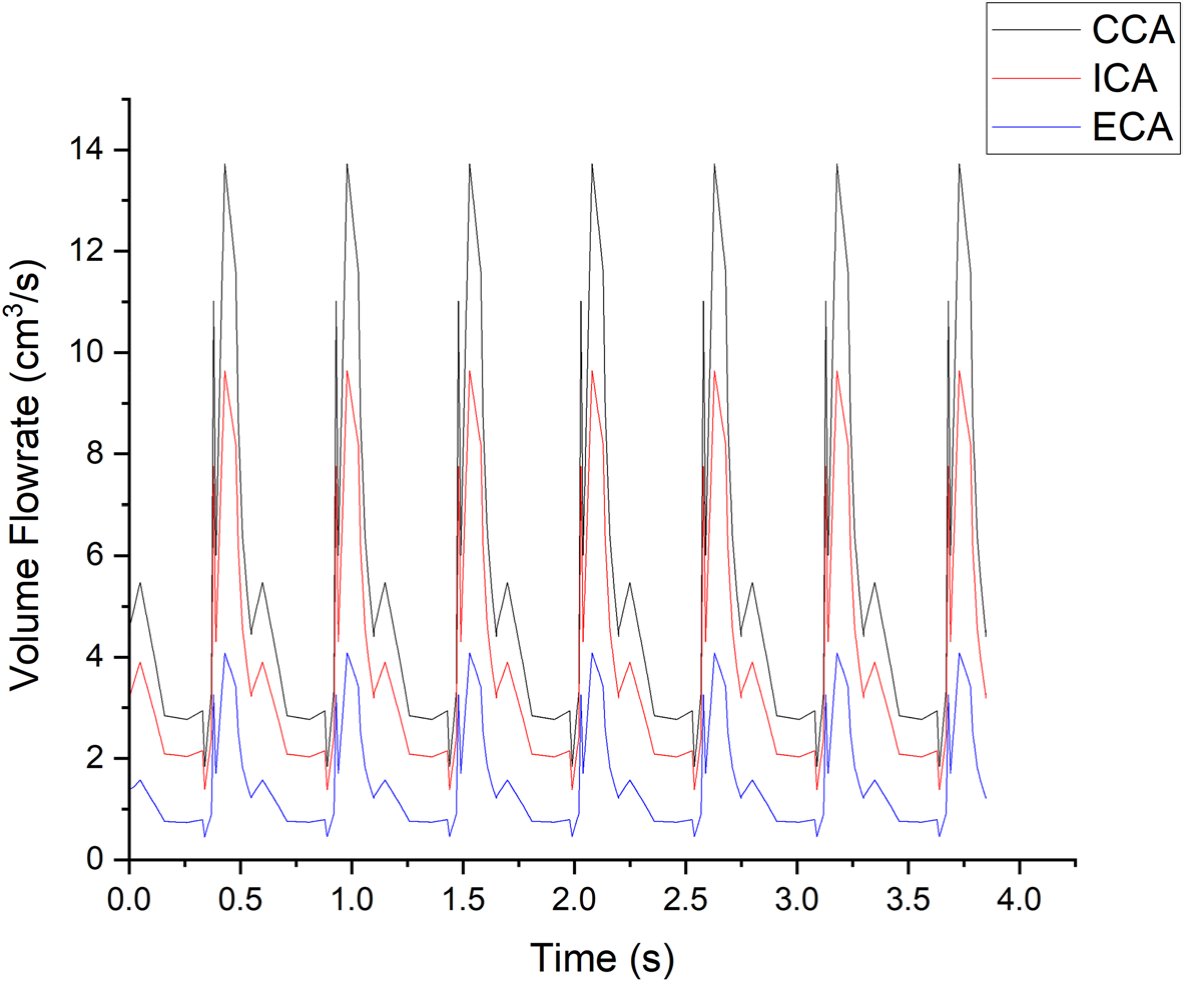
Volumetric blood flow distribution of branches of the carotid artery.

To validate the results, the resistance and compliance values for the distal and proximal segments of both ICA and ECA branches are used in a CFD simulation with the Windkessel model outlet boundary conditions. The volumetric blood flow distribution for the CCA, ICA, and ECA branches from CFD simulations for 7 cardiac cycles is shown in Fig. 4. The average blood volume flow rates for the entire 7 cardiac cycles from CFD simulations for the CCA, ICA, and ECA branches are 5.35 cm^3^/s, 3.83 cm^3^/s, and 1.52 cm^3^/s, respectively. The calculated blood volume flow rate split between ICA and ECA with respect to CCA from this study is 71.55% and 28.45%, respectively. When comparing the blood flow split ratio results to clinical data regarding the blood flow splits between ICA and ECA, it is found to be a close match. The average blood flow split between ICA and ECA calculated based on the clinical results from study ^82^ is 70.55% and 29.45%, respectively. The error percentage between the CFD results in this study and the calculated flow rate split based on clinical results from^82^ is estimated as  − 1.39% and 3.39% for ICA and ECA branches, respectively.

**Figure 5:**
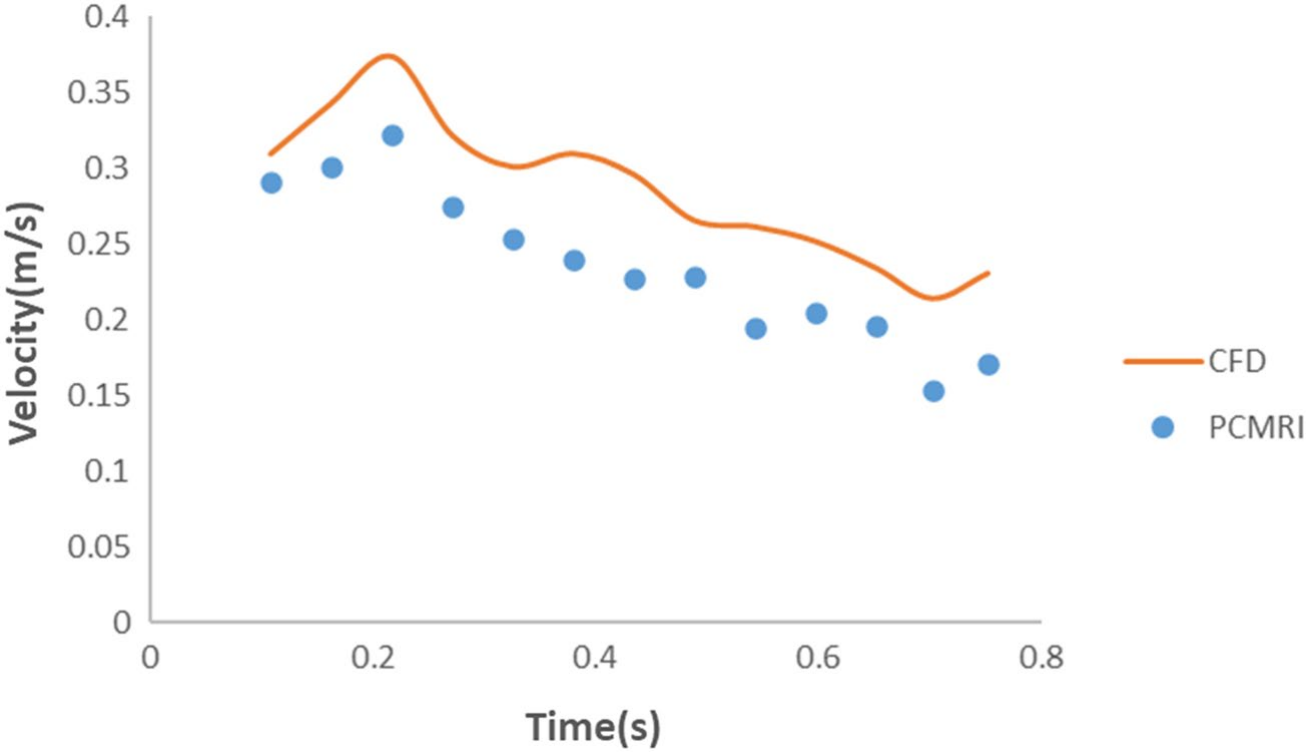
Average velocity distribution at ACA of patient specific internal carotid artery.

**Figure 6:**
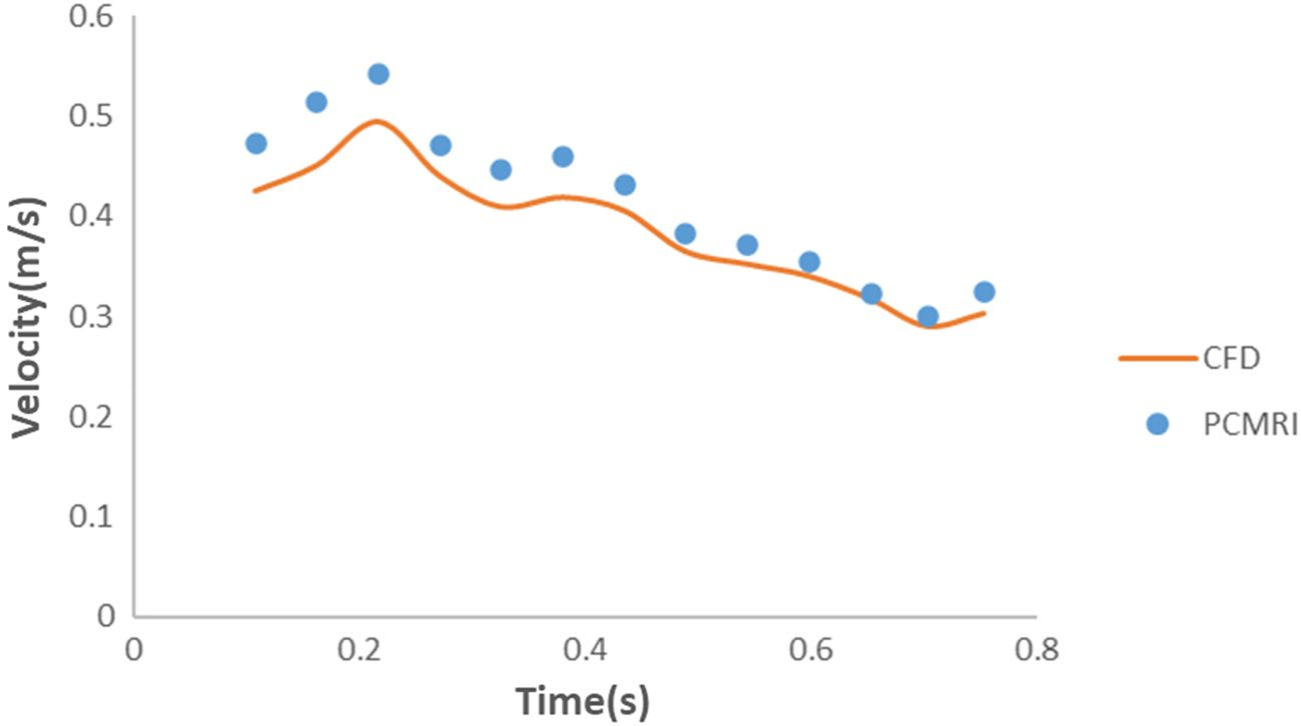
Average velocity distribution at MCA of patient specific internal carotid artery 1.

In the second validation study, the resistance and compliance values for the outlets (Ophthalmic artery, ACA, M1, and M2) were determined based on the values provided in Tables 1, 2, 3. The velocity of blood flow in the ACA branch was measured in four adjacent planes following the ICA bifurcation into the MCA and ACA. The area-weighted average of the velocity from these four planes over five cycles was calculated and depicted in Fig. 5. Similarly, the area-weighted average of blood velocity from the MCA branch was averaged over five planes and shown in Fig. 6. The measurement planes for both ACA and MCA in the CFD simulations corresponded to the same planes where the PCMRI data of the patient were collected. Figures 5 and 6 compare the velocity calculated in these simulations to the velocity obtained from the PCMRI data of the patients. From Fig. 5, it can be observed that the velocity values calculated for the ACA branch in the CFD simulations were slightly overestimated compared to the PCMRI values, with an average velocity difference of 0.051 m/s. The maximum velocity difference was 0.07 m/s at time instance 0.381 s, while the minimum velocity difference was 0.019 m/s at time instant 0.109 s. For the MCA branch, Fig. 6 demonstrates a better match between the velocity values obtained from CFD simulations and the PCMRI data. In this case, although the CFD velocity values were slightly underestimated in the first half of the cardiac cycle, they exhibited similar values to the PCMRI data in the second half of the cycle, with an average difference in velocity of 0.029 m/s for the full cycle. The maximum velocity difference was 0.064 m/s at time instance 0.163 s, while the minimum velocity difference was 0.004 m/s at time instance 0.653 s.

## Discussion

Peripheral resistance of the distal vascular elements in the cerebrovascular system is very important because it wields a lot of influence on determining the flow partition at ECA and ICA of the carotid bifurcation. It also has a significant effect on the pressure and flow distribution inside the arteries^83^. Similarly, arterial compliance is an important parameter in the application of outlet boundary conditions. It is reported that arterial compliance has a strong influence on the estimation of wall shear stress particularly in flow separation regions and recirculation zones. The compliance property of the artery wall stretches the vessels continuously which leads to changes in the wall shear stress distribution^84^. Thus the incorporation of resistance and compliance is essential for obtaining accurate CFD simulation results.

In this study, the analytically calculated values for resistance and compliance for the distal branches of the carotid artery make it easier to incorporate the Windkessel model outlet boundary conditions into the CFD domain. Results show that the distal segment of the ICA and ECA branch has more resistance compared to the proximal segments because of the more constricted geometrical appearances in the distal segments of these branches. In the case of the ICA branch, of the major branches, the Ophthalmic artery offers the most resistance but considering its geometrical size and location it might not have much influence in deciding the hemodynamics of the carotid artery. The MCA and ACA being the largest and terminal distal branches of the ICA segment exert a huge influence in deciding the blood flow characteristics in the carotid artery. Results show that ACA contributes more resistance to the blood flow when compared to the MCA branch, yet for compliance, MCA has more compliant features compared with ACA.

Amongst the segments of ACA, the A5 segment provides more resistance to flow whereas the A1 segment provides the least resistance. Regarding the MCA segment, M4 shows the most resistance whereas the M1 segment provides the least. This indicates that distal segments become narrower as they are positioned away from the proximal segment of the carotid artery. With reference to compliance, proximal segments are more compliant compared to the distal segment. A1 segment of ACA is more compliant than the A5 segment, and similarly, the M1 segment is more compliant compared to the M4 segment. In the case of the ECA segment, the Posterior Auricular Artery exhibits the highest resistance and lowest compliance whereas the Maxillary Artery has the lowest resistance and highest compliance. Consequently, the results in this study give more opportunity to understand the distal vascular characteristics of the carotid artery branches.

The CFD simulations and clinical validation carried out in this study play a crucial role in determining the applicability of the findings to research aimed at understanding the hemodynamics of the carotid artery. The validation process involved two distinct approaches. Firstly, a simplified geometry of the carotid artery was utilized, relying on generalized clinical data to focus on the flow split ratio between the ICA and ECA. Secondly, a patient-specific geometry and clinical data were employed, with a particular emphasis on the velocity profiles in the distal sections of the ICA, such as the ACA and MCA.

As discussed in the results section, the simulations performed on the simplified geometry yielded flow partition values of 71.55% and 28.45% for the ICA and ECA branches, respectively, which closely aligned with the clinical flow partition results, exhibiting error percentages of  − 1.39% and 3.39% for the ICA and ECA branches, respectively. It is important to note that the study was conducted using a healthy carotid artery, and the clinical validation data was also obtained from healthy individuals. The flow split ratio provides insights into the likelihood of downstream plaque or atherosclerotic vessels, with deviations from the established range indicating the presence of such conditions. Moreover, if the flow splits calculated from MRI differ from the results of this study, it may indicate the likelihood of atherosclerotic vessels.

The results from the patient-specific studies, focusing on the distal branches of the ICA, demonstrate that the use of analytically calculated values for the Windkessel parameters in this study yields matching velocity profiles when compared to PCMRI data. The average velocity difference for the ACA and MCA, compared to PCMRI data, is 0.05 m/s and 0.029 m/s, respectively. Although slight differences in velocity values may be observed at certain time instances, further iterations can yield matching velocity profiles, facilitating subsequent CFD simulations. It is important to note that this study does not expect a perfect match with patient-specific clinical results but rather offers a pathway for easier iterations to align with clinical data for any patient-specific geometries. Therefore, the CFD results from this study indicate that the analytically calculated values of the Windkessel parameters for the distal branches of the carotid artery can be utilized in studies focusing on distal branches, such as the MCA and ACA. These values of the Windkessel parameters, including resistance and compliance, can serve as initial values for iterations when aiming to align hemodynamic parameters obtained from CFD studies with clinical data. This helps reduce the time spent on iteration processes. Furthermore, this study provides resistance and compliance values for the major sub-branches and distal sections of the ACA and MCA, opening doors for researchers in this field to explore the hemodynamics of the distal cerebral vascular system. Consequently, researchers can eliminate numerous erroneous assumptions concerning outlet boundary conditions in these branches, leading to more accurate investigations.

## Limitations

Although this study made the best effort to identify the distal resistances at ACA and MCA by incorporating all available information on the dimensions of distal arteries from previous works, we acknowledge that the lack of detailed information on the microvasculature dimensions in our study is a universal limitation of all CFD studies investigating strokes in the cerebral arteries. Nonetheless, with the advancements in high-resolution medical imaging techniques, we believe that our paper can serve as a starting point for exploring additional resistances and compliances further downstream of ACA, MCA, and ECA in future studies, ultimately improving the accuracy of resistance and compliance calculations.

## Conclusion

Outlet boundary conditions are a very important parameter in determining the accuracy of CFD simulations. The Windkessel model outlet boundary condition is a prominent outlet boundary condition which can incorporate the distal vascular characteristics of the carotid artery into the CFD domain. The Windkessel parameters for the distal branches of the carotid artery calculated in this study through the analytical method are very relevant in obtaining more accurate CFD results. As well, the outcomes of this study give an easier option to establish the resistance and compliance of the distal branches of carotid artery. This is otherwise a tedious, time-consuming, and high computational cost operation that needs various iterations till matching with clinical results is achieved. The validation of the CFD results in this study against the clinical data confirms the relevant applicability of our findings to future studies aiming to comprehend the hemodynamics of cerebral arteries.

## Data Availability

The datasets generated during and/or analysed during the current study are available from the corresponding author on reasonable request.
